# Review of "Dynamics of the Vascular System" by John K-J Li. Series on Bioengineering & Biomedical Engineering – Vol. 1

**DOI:** 10.1186/1475-925X-4-13

**Published:** 2005-02-28

**Authors:** Michael Anbar

**Affiliations:** 1 University at Buffalo, Buffalo, NY, USA

"Dynamics of the Vascular System" is a new book by one of the world's greatest experts on bioengineering aspects of hemodynamics. It provides an excellent elementary introduction to this topic.

Following an illustrative historical introduction, the author briefly reviews vascular physiology. This is followed by the basics of fluid mechanics as an introduction to the hemodynamics of large arteries. A dedicated chapter illuminating the dynamic consequences of vascular branching follows this chapter. This is the field to which Professor Li has made his substantial contributions.

The following chapters cover the venous system and microcirculation. Finally the book reviews measuring techniques used to study hemodynamic behavior.

The author suggests this volume to be "a companion" to his own treatise "The Arterial Circulation" [1]. For those familiar with the latter book I must compare the two.

While the new book adds useful information on the venous system and on microcirculation, a topic that has been neglected in classical treatments of hemodynamics, this new volume is substantially less comprehensive in most topics covered in both. Also its index is regretfully, substantially less detailed. Little new has been published in this field since the first book was published in 2000, with the notable exception of Zamir's book "The Physics of Pulsatile Flow" [2]. The reader is advised, therefore, to consider this book as a supplement rather than a companion to the former.

In brief, this book, which is more affordable than its predecessor, should be regarded as a good introduction to the topic, to be used primarily by bioengineering students, rather than an updated authorative text by its erudite author.
